# Characterization of Dark Septate Endophytic Fungi and Improve the Performance of Liquorice Under Organic Residue Treatment

**DOI:** 10.3389/fmicb.2019.01364

**Published:** 2019-06-18

**Authors:** Chao He, Wenquan Wang, Junling Hou

**Affiliations:** ^1^Institute of Medicinal Plant Development, Chinese Academy of Medical Sciences and Peking Union Medical College, Beijing, China; ^2^School of Chinese Pharmacy, Beijing University of Chinese Medicine, Beijing, China

**Keywords:** dark septate endophytes, plant performance, soil properties, organic residues, liquorice, symbiosis

## Abstract

Dark septate endophytic (DSE) fungi is a diverse group of Ascomycetes fungi that colonize the plants roots, and may facilitate plant growth and fitness, however, their ecological roles need further clarification. This study aimed to evaluate the growth promoting effects of DSE fungi in a medicinal plant, liquorice (*Glycyrrhiza uralensis*), under additional organic residues. First, we isolated, identified and characterized, two DSE fungal species (*Acrocalymma vagum* and *Paraboeremia putaminum*) harboring inside the roots of liquorice growing in arid areas of China. Second, we examined the performance and rhizosphere soil parameters of liquorice plants inoculated with these fungi under additional sterilized organic residues and unsterilized organic residue (containing *Trichoderma viride* population) in a growth chamber. The results showed that two DSE strains could effectively colonize plant roots and formed a strain-dependent symbiosis with liquorice. DSE inoculation alone increased the plant biomass, and glycyrrhizic acid and glycyrrhizin content. It also improved the root system and N and P absorption by plants, consequently depleting these macronutrients in the soil. Residues alone increased soil organic matter, available phosphorus (P), and available nitrogen (N) content, and plant biomass, N, P, glycyrrhizic acid, and glycyrrhizin content. Mantel test and structural equation model (SEM) analysis demonstrated that DSE associated with residues significantly positively influenced soil organic matter, available P and available N, and plant biomass, glycyrrhizin, N, P, and root surface area. Variation in plant growth and glycyrrhizic acid and glycyrrhizin accumulation can be attributed to the effects of DSE inoculation. DSE associated with residues exhibited a general synergistic effect on the growth and accumulation of glycyrrhizic acid and glycyrrhizin of liquorice. We demonstrate for the first time, two DSE fungi in the liquorice roots that have potential use as promoter for the cultivation of medicinal plant.

## Introduction

Liquorice (*Glycyrrhiza uralensis* Fisch.), belonging to Fabaceae, is an herbaceous perennial medicinal plant widely grown in the world (Liu et al., 2007). It is officially included in Chinese Pharmacopeia, and it possesses several pharmacological effects and biological functions owing to the presence of components such as glycyrrhizin and glycyrrhizic acid (Jassal et al., 2015; Shen et al., 2015). Liquorice also has been widely used as a food supplement and cosmetic additive (Kao et al., 2014). In addition, since liquorice is well adapted to low-fertility soil and arid conditions, this plant is expected to be widely used for ecological restoration in degraded ecosystems and arid areas (Liu et al., 2007; Chen et al., 2017).

Liquorice is presently cultivated to meet the increasing demand for liquorice in the herbal industry. However, nutrient deficiency and drought stress affects the quality and productivity of cultivated liquorice. To seek and apply effective methods for high-yield and high-quality plant material, recent studies have focused on investigating soil microorganisms that are beneficial to the medicinal plants (Zubek and Błaszkowski, 2009; Zhu et al., 2015). The beneficial effects of root-associated fungi such as arbuscular mycorrhizal fungi (AMF), dark septate endophytic (DSE) fungi, and *Trichoderma* spp. (Zubek and Błaszkowski, 2009; Zhang et al., 2010; Guimarães et al., 2014) include the following: improve the growth, photosynthetic activity, and P content of plants; act antagonistically toward soil-borne fungal pathogens; and alter the concentration of secondary metabolites in plants (Weisany et al., 2016; Lima et al., 2017; Vergara et al., 2017).

Dark septate endophytic fungi are a diverse group of fungi with darkly pigmented and septate hyphae and are frequent intracellular root associates of plants (Piercey et al., 2004). They colonize the cortical cells and intercellular regions of the roots and occasionally form densely septate intracellular structures (microsclerotia) (Jumpponen and Trappe, 1998). In contrast to the vast information on AMF, information on the role of DSE fungi in the ecosystem is limited. A meta-analysis has showed that the relationships between host plants and DSE fungi range from symbiotic to parasitic associations. The effects of DSE fungi on host plants have been reported to vary and depend on the host-symbiont combination (Jumpponen, 2001; Mayerhofer et al., 2013). Previous studies have showed that DSE fungi inoculation not only enhances the growth of medicinal plants but also improves the production and quantity of compounds in plants (Wu and Guo, 2008; Zhang et al., 2012). Indeed, several DSE fungi act as plant growth promoters by facilitating C, N, and P uptake (Della Monica et al., 2015; Surono and Narisawa, 2017), and by protecting plants against biotic (pathogen) and abiotic (heavy metal, elevated CO_2_, and drought) stresses (Likar and Regvar, 2013; Santos et al., 2017; Surono and Narisawa, 2017). In addition to DSE fungi, the role of *Trichoderma* spp. as biological control agents and/or as biofertilizers in agricultural and horticultural systems has also been investigated (Contreras-Cornejo et al., 2018). *Trichoderma* spp. also improve the growth of plants via numerous mechanisms, mainly by solubilizing soil nutrients (Yadav et al., 2009; Kapri and Tewari, 2010), and increasing root length, secondary root number, and phytohormones, such as indole acetic acid, cytokinin, gibberellins, and zeatin (Gravel et al., 2007; Contreras-Cornejo et al., 2016). Hence, there is a need for research on using these beneficial fungi to promote the plant growth and medicinal quality of medicinal plants.

Liquorice residues are cellulose-rich by-products obtained during the manufacture of Chinese medicine and other related products. These residues after proper microbial fermentation act as good organic amendments and can be used as a soil conditioner or organic fertilizer for plants (Rashid et al., 2016; He et al., 2017; Vimal et al., 2017). As *Trichoderma* spp. are cellulolytic fungi, they are used to degrade liquorice residue, enabling easy absorption of macromolecular carbohydrates in the residue, which are not easily absorbed by plants, as small molecular nutrients (Mutschlechner et al., 2015). In turn, the nutrients released in the degradation process can also be utilized for the survival of *Trichoderma* spp. (Chovanec et al., 2001). Moreover, plant–microbe–manure tripartite interactions may play vital roles in sustainable agricultural management under stressed condition because such associations play an important role in improving the performance of crops (Yang et al., 2015).

In this study, we aimed to evaluate whether DSE fungi from arid habitats could successfully colonize the roots of liquorice seedlings grown in sandy soil under greenhouse conditions and enhance plant growth under sterilized organic residues and unsterilized organic residue (containing *Trichoderma viride* population) treatment. Therefore, we first investigated and isolated DSE strains in the roots of liquorice. Second, we conducted an inoculation experiment to evaluate the growth of liquorice response to these host DSE fungi. We addressed the following questions: (1) What is the characterization of DSE fungi in the roots of liquorice in the arid areas of north China? (2) Do these DSE fungi act as host colonizer and affect the growth and nutrient uptake of liquorice under artificial culture conditions? (3) Whether residue availability affects the symbiosis-dependent benefits?

## Materials and Methods

### Fungal Materials

Root samples of liquorice were collected from the Anguo Medicine Planting Site (115°20′E, 38°24′N) in Hebei Province, China. Root samples were washed in deionized water, sterilized in 75% ethanol for 5 min and then in 10% sodium hypochlorite for 5 min, rinsed three times in deionized water, dried on sterile filter paper, and placed in Petri dishes in potato dextrose agar (PDA) culture medium with antibiotic supplements (ampicillin and streptomycin sulfate). The dishes were incubated at 27°C in the dark and were observed daily (Zhan et al., 2015). To confirm that the isolates were true endophytes, 200 μL of the distilled water left in the final step were coated on PDA medium as a contrast. Mycelium growing from the cut ends of root segments were transferred to new PDA plates and kept in the dark at 27°C before performing microscopic observations and measurements. Moreover, each isolate had three replicates that were cultured at 10°C for 2 months to induce sporogenesis (Knapp et al., 2015).

Fresh mycelia (approximately 50 mg) were scraped from the surface of PDA plates, and DNA was extracted using a genomic DNA extraction kit (Solarbio, China). Two primers, ITS4 (5′-TCCTCCGCTTATTGATATGC-3′) and ITS5 (5′-GGAA GTAAAAGTCGTAACAAGG-3′), were used for all isolates. PCR was performed in 40 μL volumes containing 7 μL of fungal genomic DNA, 1 μL of each primer, 20 μL of 2 × Es Taq Master Mix, and 11 μL of ddH_2_O. PCR cycling was performed in a Life ECOTM system (BIOER, China) using the following program: initial denaturation at 94°C for 5 min; followed by 35 cycles of 94°C 1 min, 55°C 1 min, 72°C 1 min; and a final incubation at 72°C for 10 min (Xie et al., 2017). The PCR products were purified and sequenced, and then sequences were deposited in GenBank under the accession numbers MK392024 (DSE1) and MK601233 (DSE2). The sequences were aligned via MUSCLE with G-block curation (Edgar, 2004), and the tree was inferred by the maximum likelihood (ML) method via PhyML (Guindon and Gascuel, 2003) implemented at the phylogeny.fr website (Dereeper et al., 2008). Branch-support values of the phylogenetic tree were estimated using the approximate likelihood-ratio test (Anisimova and Gascuel, 2006) with the SH-like option.

*Trichoderma viride* for solid state fermentation was isolated from the rhizosphere soil of liquorice in the planting base of Karamay City, Xinjiang, China and identified based on morphological characters and ITS phylogeny; its ITS sequence is available at GenBank under the accession number MK396066. The fungal strain is deposited in the culture collection of the Laboratory of Endangered Species Breeding Engineering, Institute of Medicinal Plant Development, Chinese Academy of Medical Sciences and Peking Union Medical College, Beijing, China.

### Organic Residue and Plant Cultivation

Liquorice (*G. uralensis* Fisch.) was chosen as a host plant in this study mostly for its important role in pharmacology and vegetation restoration. Seeds of liquorice were provided by China National Traditional Chinese Medicine Corporation, Beijing, China, and stored at 4°C. Liquorice residues (after acid extraction) were obtained from Chinese Natural Plant Products. Residue were ground in a milling machine (SFM-1; Hefei, China), passed through a sieve (size 80), and stored in a sealed pot at 25°C. Solid state fermentation of the residue by *T. viride* was carried out according to the method of Muhammad et al. (2014). After 13 days, the fermented residue with 40.08% optimal cellulose degradation rate was ready for use as organic residue. The pot-based plant culture involved growing liquorice plant in a 1:1 mixture of farmland soil and washed river sand. The organic residue was ground and passed through a 2-mm sieve, and then autoclaved for 90 mm at 121°C. The mixed substrate contained 21.57 mg/g organic matter, 85.19 mg/kg available nitrogen (N), and 7.90 mg/kg available phosphorus (P).

### Experimental Procedure

The experiment was conducted in a growth chamber in a completely randomized factorial design (3 inoculation treatments × 2 residue conditions × 4 inoculation plus residue treatments) with 5 replicates. The inoculation treatments included inoculation with DSE1 and DSE2 and a non-inoculated control. The residues used included both sterilized (after autoclaving for 90 min at 121°C) and unsterilized residues. Liquorice seeds were surface sterilized by dipping them in 70% ethanol for 3 min and 2.5% sodium hypochlorite for 10 min, and then rinsing them three times in sterile distilled water. The sterilized seeds were placed on aseptic water agar medium (agar 10 g/L) for germination at 27°C. Following pregermination, each seedlings were transplanted into sterile glass bottles (6 cm diameter, 12 cm height) containing sterile culture substrate (500 g). Two seedlings were planted in each sterile microcosm. For DSE-inoculated treatments, four 5 mm plugs excised from an edge of an actively growing colony on culture medium were inoculated at a 1 cm range close to the roots of liquorice seedlings. For residue treatments, 10 g of sterilized or unsterilized residue and 500 g of sandy soil are well blended as growing substrate. The control treatments were inoculated with plugs excised from the sterile medium without fungus and residue. All inoculation and residue addition processes were performed on an ultra-clean workbench. All the experimental bottles were kept in a growth chamber with a 14 h/10 h photoperiod, a temperature of 27°C/22°C (day/night), and 60% mean air relative humidity. The duration of the experiment was 3 months.

### Plant Growth Parameters

At the end of the experiment, shoots and roots from each bottle were separately harvested, and roots were gently washed with tap water to remove the sand. Individual root sections were floated in water at approximately 1-cm depth in a plexiglass tray and scanned using a desktop scanner (EPSON Perfection V800 Photo, Japan). The total length, surface area, average diameter, and root branch number were determined using the WinRHIZO image analysis system (Chen et al., 2012). The shoot and root biomass were measured after drying the samples in an oven at 70°C for 48 h. Thereafter, the dried shoot and root materials were ground to a powder to measure the concentration of nitrogen (N) and phosphorus (P). Soil samples were air-dried under cool conditions (15°C–25°C) and stored in plastic bags at 4°C until analysis for soil physicochemical properties.

### DSE Root Colonization

Root colonization by DSE isolates was evaluated using the method described by Phillips and Hayman (1970). The sampled roots were cleared with 10% KOH in a water bath at 100°C for 60 min and then stained with 0.5% (w/v) acid fuchsin at 90°C for 20 min. Overall, 20 randomly selected 0.5 cm long root segments per sample were placed on slides and observed under an optical microscope.

### Quantification of Active Ingredients in the Roots

The dried root samples of liquorice plants were ground into powder with a mortar and pestle and passed through a 40-mesh sieve. Approximately 0.05 g sample was weighed accurately and extracted in 10 mL methanol/water (70:30) for 30 min in an ultrasonic bath at 25°C. The extract solution was cooled to 25°C and filtered through a 0.45 μm filter. A 10-μL aliquot of the filtrate was subjected to separation by high–performance liquid chromatography using a reverse phase C_18_ symmetry column (4.6 mm × 250 mm, pore size 5 μm; Waters Corp., Milford, MA, United States). The mobile phase comprised a gradient of deionized water:phosphoric acid (100:0.05, v/v) and acetonitrile. The separation was conducted in the gradient elution mode (Supplementary Table S1) at 25°C with a flow rate of 1.0 mL/min. The eluted compounds were detected spectrophotometrically at 237 nm using a 2998 PDA photodiode array detector. Standard substances of glycyrrhizic acid and glycyrrhizin were purchased from China National Institutes for Food and Drug Control. Their stock solutions were diluted with 70% aqueous methanol to appropriate concentrations for calibration purpose (Zhang et al., 2013).

### Determination of Mineral Content in Soil and Plant Tissue Samples

The dried plant tissue or soil sample (0.2 g) was digested in 10 mL of mixture of perchloric acid (12.7 mol/L), sulfuric acid (18 mol/L), and water at a ratio of 10:1:2 using the Mars 6 microwave reaction system (CEM Corporation, Matthews, NC, United States) until a clear liquid was obtained. The content of total N and P in the samples was analyzed by Kjeldahl method and vanadium molybdate blue colorimetric method, respectively (Bao, 2000). The content of soil organic matter, available N, and available P was quantified by oxidization with dichromate in the presence of sulfuric acid (Rowell, 1994), alkaline hydrolysis-diffusion method, and chlorostannous- reduced molybdophosphoric blue method (Olsen et al., 1954), respectively.

### Statistical Analyses

The effects of DSE, residue, and their interaction on the measured variables were assessed by the two-way analysis of variance (*P* < 0.05). The statistical significance of the results was determined by performing Duncan’s multiple-range tests (*P* < 0.05). Mantel test and the structural equation model (SEM) were used to test the effects of DSE species, residue, and soil parameters on the growth and active ingredients of liquorice seedlings using R-3.2.2 packages ecodist (Goslee and Urban, 2007) and AMOS 21.0 (maximum likelihood). Variation partitioning was performed to estimate the size of effect that each factor has on plant growth and active ingredients. SPSS 21.0, Canoco 4.5, RStudio packages vegan (Borcard et al., 2011), and Kaleida Graph 4.5 were used for statistical analyses and plotting.

## Results

### Characterization and Identification of DSE Fungi in the Roots of Liquorice

**FIGURE 1:**
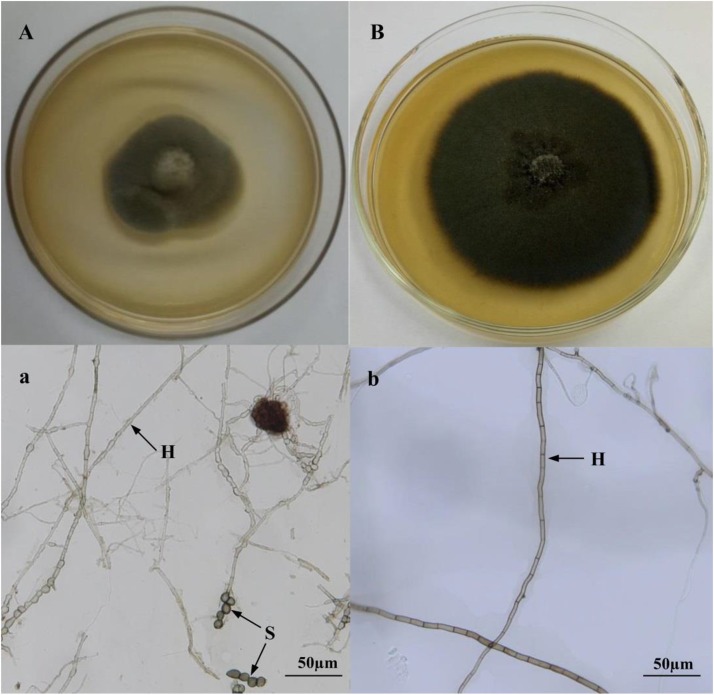
Colonies of endophytic fungi isolated from the roots of liquorice **(A,B)**. Microscopic morphology of endophytic fungi **(a,b)** (bars = 50 mm). **A,a**: *Acrocalymma vagum*; **B,b**: *Paraboeremia putaminum*. Arrows indicate: H, DSE hyphae; S, DSE spores.

Two DSE colonies isolated from liquorice roots were gray to dark-brown, and are illustrated in Figure 1. DSE1 inoculation media produced chlamydospores (Figure 1A), whereas conidia or reproductive structures were not observed in DSE2 media in culture at 27°C (Figure 1B). Growth curves of the isolated DSE strains were measured by recording colony diameters every day for 2 weeks. The growth curves of isolates DSE1 and DSE2 were linear, with average growth rates of 3.35 and 3.18 mm d^–1^, respectively.

**FIGURE 2:**
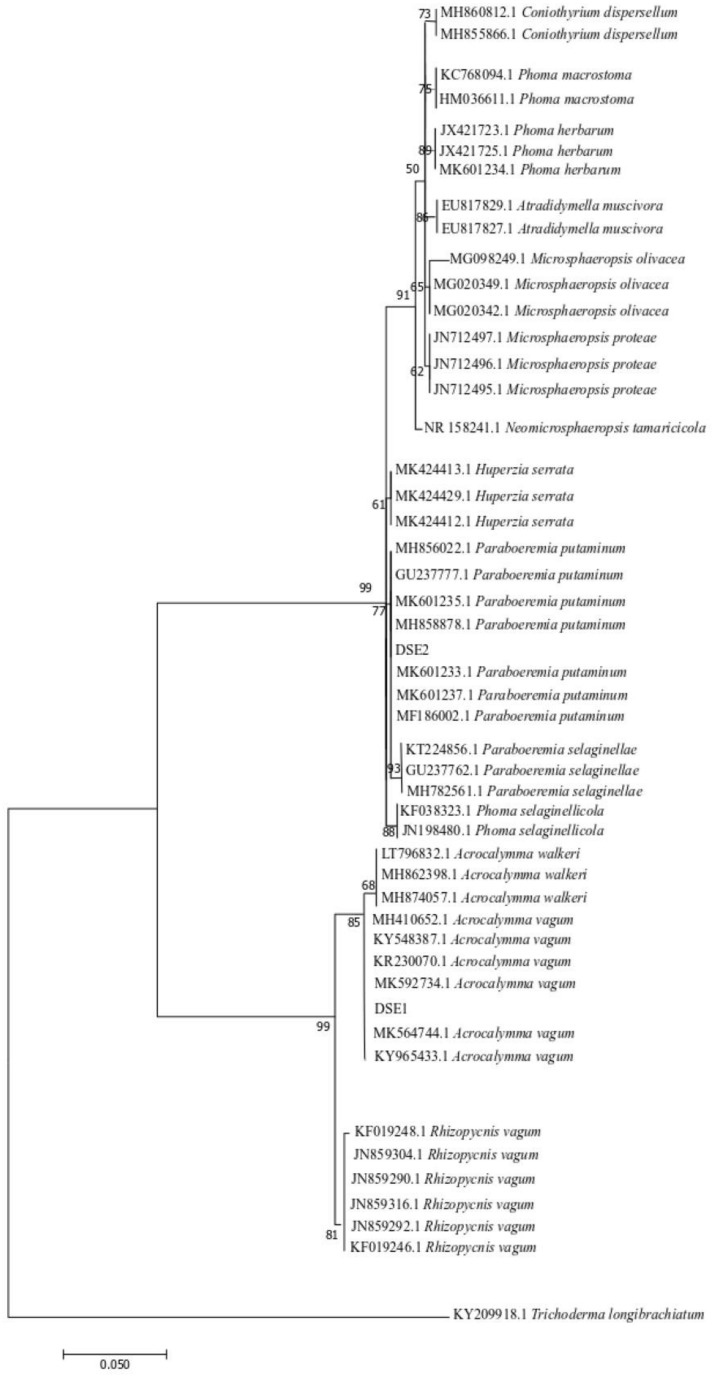
Maximum parsimony tree generated from ITS (ITS4 and ITS5) sequences of the isolate strains and their closest matches, followed by GenBank accession number.

The phylogenesis of the ML tree based on the ITS4-5.8S-ITS5 rDNA is shown in Figure 2. Based on the morphological characteristics and molecular phylogenetic analysis, DSE1 and DSE2 were identified as *Acrocalymma vagum* and *Paraboeremia putaminum*, respectively.

**FIGURE 3:**
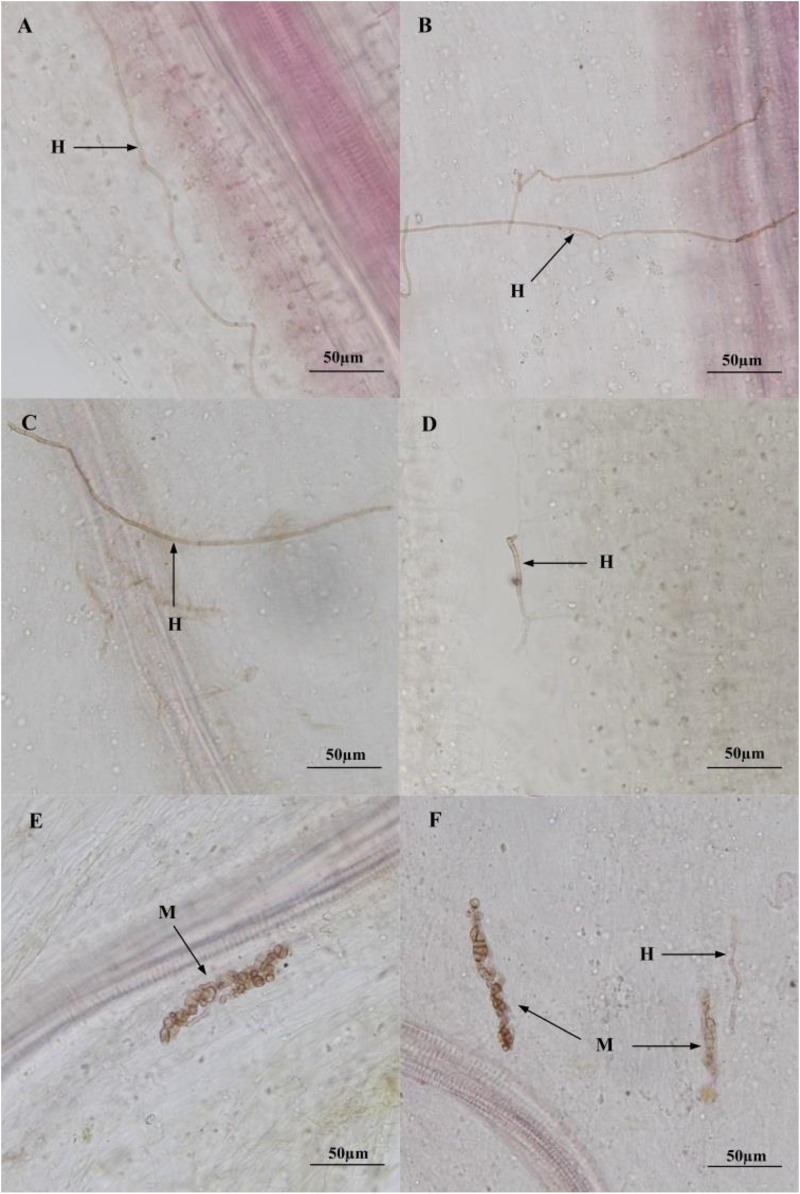
Colonization of dark septate endophyte (DSE) strains in the roots of liquorice plant 3 months after inoculation. **(A–F)** Indicate roots inoculated with *A. vagum*, *P. putaminum*, *A. vagum* + sterilized residue, *P. putaminum* + sterilized residue, *A. vagum* + unsterilized residue, *P. putaminum* + unsterilized residue, respectively. Arrows indicate: H = DSE hyphae, M = DSE microsclerotia.

After harvest, no DSE structures were observed in the roots of control plants regardless of the residue treatment, whereas the presence of DSE hyphae and microsclerotia was confirmed in the stained root segments of inoculated plants (Figure 3).

### Root Morphology Traits

**FIGURE 4:**
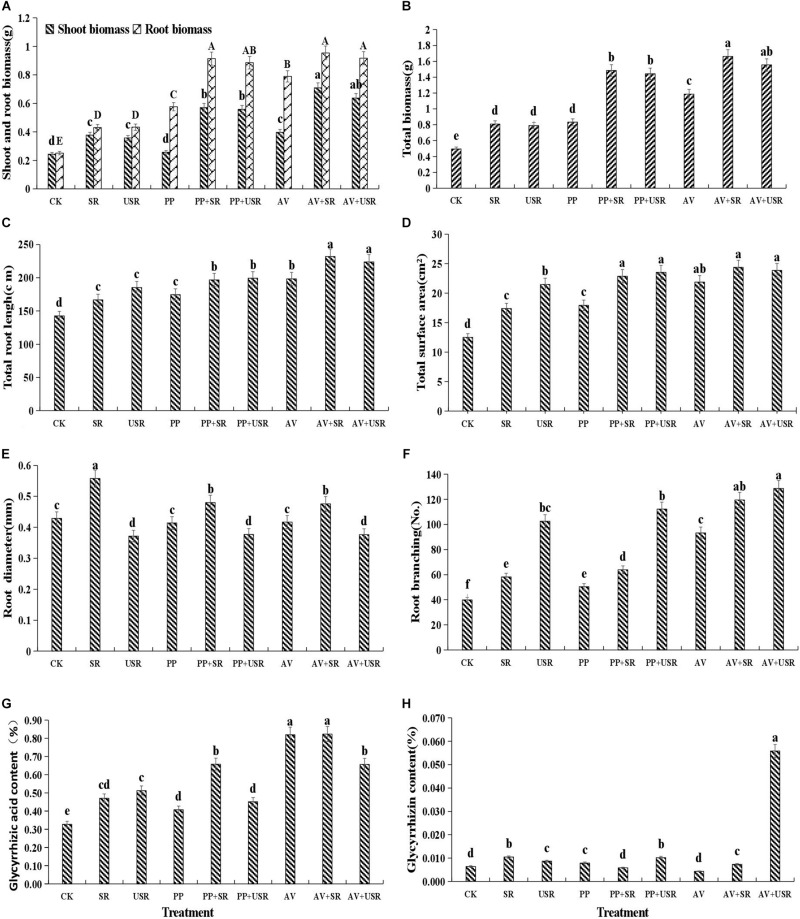
Effects of dark septate endophyte (DSE) inoculation and residues treatment on the growth and active ingredient content of liquorice plants. The error bars represent the standard error (SE). Different letters above the error bars indicate significant difference at *P* < 0.05 by Duncan’s multiple-range tests. CK indicates non-inoculated plants and no residues. SR, sterilized residue. USR, unsterilized residue. AV, *A. vagum*. PP, *P. putaminum*. AV+SR, *A. vagum*+sterilized residue. AV+USR, *A. vagum* + unsterilized residue. PP+SR, *P. putaminum*+sterilized residue. PP+USR, *P. putaminum*+ unsterilized residue. The shoot biomass and root biomass of liquorice plants **(A)**, total biomass of liquorice plants **(B)**, total root length of liquorice plants **(C)**, total root surface area of liquorice plants **(D)**, root diameter of liquorice plants **(E)**, root branching of liquorice plants **(F)**, glycyrrhizic acid content of liquorice plants **(G)**, glycyrrhizin content of liquorice plants **(H)**.

Compared with that of the non-inoculated plants, DSE inoculation significantly increased the root length, surface area, and branch number, but it did not affect the root diameter. Compared with that of the treatment with no residue addition, the addition of residue significantly improved the root morphology traits. Unsterilized residue addition significantly increased the root surface area and branch number, but decreased the root diameter compared with that by sterilized residue treatment (Figure 4). DSE combined with sterilized residue significantly increased the root length, surface area and branch number, and decreased the root diameter, compared with that of the sterilized residue alone treatment. However, *P. putaminum* combined with unsterilized residue only increased the root length and surface area, and *A. vagum* combined with unsterilized residue only significantly increased the root length, surface area, and branch number, compared with that of the unsterilized residue alone treatment (Table 1 and Figure 4).

**TABLE 1:** Analysis of variance (ANOVA) for the effects of dark septate endophyte (DSE) inoculation and residue treatment (Residue) on the root morphological traits of liquorice plants.

	**Root length**		**Root surface**		**Root diameter**		**Root branch**	
	**(cm)**		**area (cm^2^)**		**(mm)**		**(No.)**	
	***F***	***P***	***F***	***P***	***F***	***P***	***F***	***P***
DSE	10.8	<0.001	30.5	<0.001	6.814	0.002	12.6	<0.001
Residue	4.4	0.017	24.8	<0.001	103.4	<0.001	14.5	<0.001
DSE × Residue	0.3	0.891	3.2	0.018	5.4	<0.001	0.8	0.510

*Significant *P*-values are in bold.*

### Plant Biomass Production

Dark septate endophytic fungi inoculation or residue addition alone significantly increased the plant biomass compared with that of the control plants. *A. vagum* significantly increased the shoot biomass, root biomass and total biomass compared with that by *P. putaminum*. However, no significant difference in shoot biomass, root biomass, and total biomass were observed between the sterilized and unsterilized residue treatments (Figure 4). The interaction of DSE and residue was significant for the shoot biomass (Table 2 and Figure 4). DSE inoculation significantly increased the shoot biomass, root biomass and total biomass compared with that in the non-inoculated plants with residue treatments. The highest biomass was observed in plants inoculated with *A. vagum* under additional sterilized residue. However, DSE inoculation did not affect plant biomass between the sterilized and unsterilized residue treatments (Table 2 and Figure 4).

**TABLE 2:** Analysis of variance (ANOVA) for the effects of dark septate endophyte (DSE) inoculation and residue treatment (Residue) on plant biomass and active ingredient content of liquorice plants.

	**Shoot biomass (g)**		**Root biomass (g)**		**Total biomass (g)**		**Glycyrrhizic acid (%)**		**Glycyrrhizin (%)**	
	***F***	***P***	***F***	***P***	***F***	***P***	***F***	***P***	***F***	***P***
DSE	85.7	<0.001	189.2	<0.001	234.5	<0.001	117.4	<0.001	446.3	<0.001
Residue	99.2	<0.001	39.8	<0.001	102.7	<0.001	19.5	<0.001	730.9	<0.001
DSE × Residue	6.1	<0.001	2.5	0.056	4.8	0.003	13.8	<0.001	452.3	<0.001

*Significant *P*-values are in bold.*

### Active Ingredient Contents

*Paraboeremia putaminum* inoculation significantly increased the glycyrrhizic acid and glycyrrhizin content, whereas, *A. vagum* inoculation only significantly increased the glycyrrhizic acid content, compared with that in the non-inoculated plants. Compared with that in plants that did not receive any residue, the addition of residue significantly increased the glycyrrhizic acid and glycyrrhizin content (Table 2 and Figure 4). The interaction of DSE and residue was significant for active ingredient content in the roots (Table 2). DSE combined with sterilized residue significantly increased the glycyrrhizic acid content and decreased the glycyrrhizin content. *A. vagum* combined with unsterilized residue increased the glycyrrhizic acid and glycyrrhizin content, but *P. putaminum* combined with unsterilized residue increased the glycyrrhizin content and decreased the glycyrrhizic acid content in the roots, compared with that in plants subjected to unsterilized residue alone treatment (Figure 4).

### Plant and Soil Mineral Nutrient Status

**FIGURE 5:**
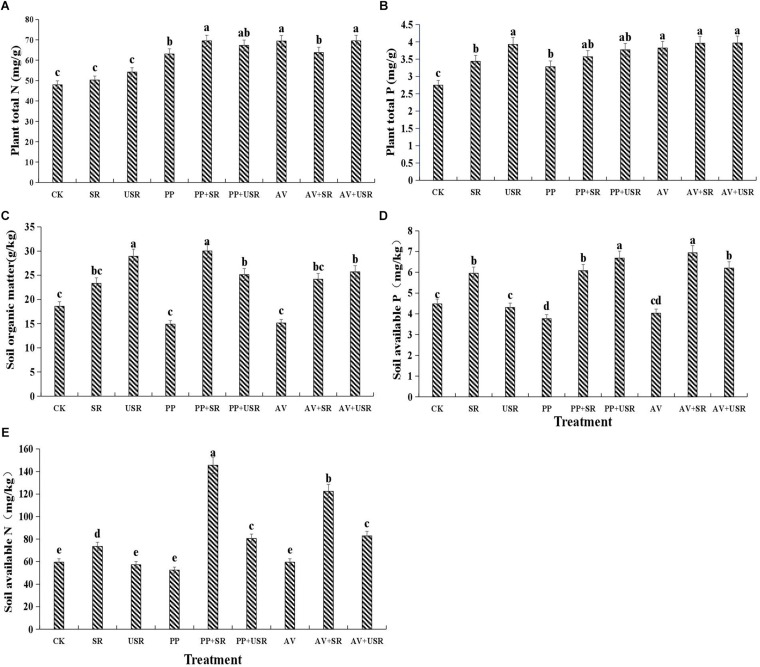
Effects of dark septate endophyte (DSE) inoculation and residues treatment on the plant and soil mineral nutrient contents of liquorice plants. The error bars represent the standard error (SE). Different letters above the error bars indicate significant difference at *P* < 0.05 by Duncan’s multiple-range tests. CK indicates non-inoculated plants and no liquorice residues. SR, sterilized residue. USR, unsterilized residue. AV, *A. vagum*. PP, *P. putaminum*. AV+SR, *A. vagum*+sterilized residue. AV+USR, *A. vagum* + unsterilized residue. PP+SR, *P. putaminum*+sterilized residue. PP+USR, *P. putaminum* + unsterilized residue. Plant total N of liquorice plants **(A)**, plant total P of liquorice plants **(B)**, soil organic matter of rhizosphere soil **(C)**, soil available P of rhizosphere soil **(D)**, soil available N of rhizosphere soil **(E)**.

In all the inoculated plants, DSE inoculation significantly increased the plant N and P content, but only *P. putaminum* significantly decreased the soil available P content compared with that in the non-inoculated plants. The addition of sterilized residue significantly increased the soil available N and available P, and plant P content, but the addition of unsterilized residue only significantly increased the soil organic matter and plant P content, compared with that in plants that received no residue (Table 3 and Figure 5). The interaction of DSE and residue was significant for plant N and soil mineral nutrient status (Table 3). For sterilized residue treatments, *P. putaminum* inoculation significantly increased the soil organic matter and available N, and plant N content, but *A. vagum* inoculation significantly increased the soil available P and available N, and plant N and P content. For unsterilized residue treatments, DSE inoculation significantly increased the soil available P and available N, and plant N content, but only decreased the soil organic matter content (Figure 5).

**TABLE 3:** Analysis of variance (ANOVA) for the effects of dark septate endophyte (DSE) inoculation and residue treatment (Residue) on the plant and soil nutrient content of liquorice plants.

	**Plant total**		**Plant total**		**Soil organic**		**Soil available**		**Soil available**	
	**N (mg/g)**		**P (mg/g)**		**matter (g/kg)**		**P (mg/kg)**		**N (mg/kg)**	
	***F***	***P***	***F***	***P***	***F***	***P***	***F***	***P***	***F***	***P***
DSE	6.5	0.004	10.8	<0.001	9.2	<0.001	16.3	<0.001	141.2	<0.001
Residue	18.8	<0.001	8.2	<0.001	205.9	<0.001	102.7	<0.001	478.7	<0.001
DSE × Residue	9.9	<0.001	5.4	0.002	20.2	<0.001	21.9	<0.001	76.6	<0.001

*Significant *P*-values are in bold.*

### Correlation Analyses

**FIGURE 6:**
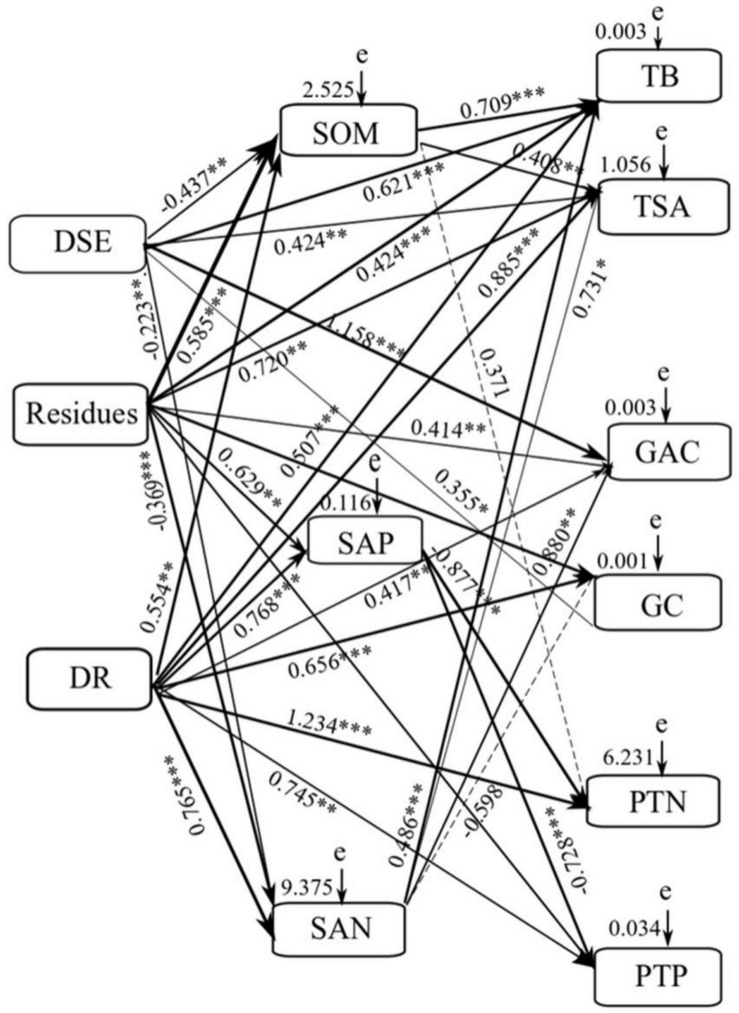
Structural equation model (SEM) showing the causal relationships among soil factors, DSE species, residues and growth indicators, active ingredient and nutrients content. The final model fitted the data well: maximum likelihood, *X*^2^ = 130.161, *df* = 21, *P* = 0.007, goodness-of-fit index = 0.741, Akaike information criteria = 156.672, and root mean square error of approximation = 0.344. Solid lines and dashed lines indicate significant and non-significant pathways, respectively. The width of the solid lines indicates the strength of the causal effect, and the numbers near the arrows indicate the standardized path coefficients (^*^P < 0.05, ^∗∗^*P* < 0.01, ^∗∗∗^*P* < 0.001). DR = combination of DSE and residues. SOM = soil organic matter. SAP = soil available P. SAN = soil available N. e = the values of residuals. TB = total biomass. TSA = root surface area. GAC = glycyrrhizinic acid content. GC = glycyrrizin content. PTN = plant total nitrogen. PTP = plant total phosphorus.

Mantel test and the SEM were used to illustrate the effects of DSE species, residue, and soil parameters on the growth parameter, active components, and nutrient content of liquorice. Mantel test showed significant relationships between DSE, residue, plant biomass, root surface area, glycyrrhizic acid, glycyrrhizin, plant N, plant P, and soil nutrient elements (Supplementary Table S2). Using the correlation coefficients (*R*-values), we used the SEM to quantify the relative effects of DSE, residue, combination of DSE and residue, soil organic matter, available P, and available N on plant biomass, root surface area, glycyrrhizic acid, glycyrrhizin, N, and P in plants (*X*^2^ = 130.161, *df* = 21, *P* = 0.007, RMSEA = 0.344, GFI = 0.741, AIC = 156.672; Figure 3). Our results revealed that DSE had significant direct effects on plant biomass, root surface area, and glycyrrhizic acid, glycyrrhizin, N, and P content. However, DSE had direct negative effects on soil organic matter and available N content. Residue had significant direct effects on soil organic matter, and plant biomass, root surface area, and glycyrrhizic acid, glycyrrhizin, and P content. However, residue had direct negative effect on soil available N. The combination of DSE and residue significantly positively influenced the soil organic matter, available P, and available N content; plant biomass; root surface area; and plant glycyrrhizin, N, and P content. Soil organic matter had significant direct effects on the plant biomass and root surface area. Soil available N had significant direct effects on the plant biomass, root surface area, and glycyrrhizic acid content. Moreover, soil available P significantly negatively affected the N and P content in plants (Figure 6).

### Variation Partitioning of Growth Parameter

**FIGURE 7:**
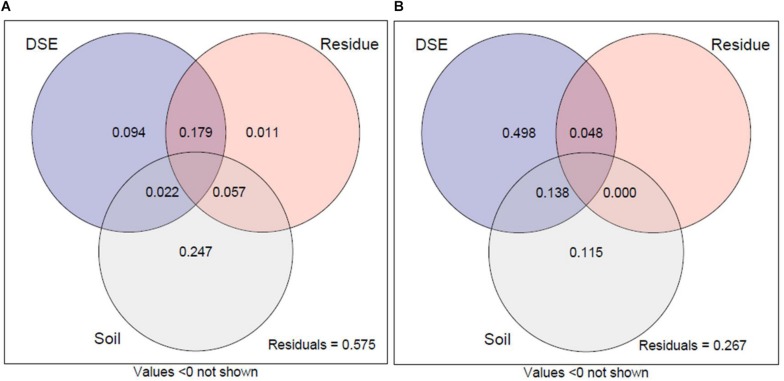
Variation partitioning of DSE species, residues, and soil mineral nutrient on plant biomass, root morphology traits, and nutrient content **(A)**. Variation partitioning of DSE species, residues, and soil mineral nutrient on active ingredient contents in roots **(B)**. DSE, DSE species; Residue, liquorice residues; Soil, mineral nutrient content (including soil organic matter, available P, and available N). Values below 0 are not shown.

Variance partitioning analysis was performed to quantify the contribution of DSE species, residue, and soil mineral nutrient status to plant growth and active ingredient content (Figure 7). The combination of DSE species, residue, and soil mineral nutrient status explained 42.5% of the variation in the growth parameter of liquorice (Figure 7A) and 73.3% of the variation in active ingredient content (Figure 7B). The interpretation of soil mineral nutrient status on plant growth accounted for 24.7% and the variation in growth index explained by DSE and residue accounted for just 9.4% and 1.1%, respectively. The interactive effects of DSE and residue accounted for 17.9%, which demonstrated the positive interactive influence of DSE in combination with residue on liquorice growth. For active ingredient content, the main variation is attributed to the pure effect of DSE species, which accounted for 49.8% individually, whereas the variation in residue on active ingredient content was mostly dependent on the simultaneous effects of DSE and residue, which accounted for 4.8%. The pure variability accounted for by the soil mineral nutrient status on active ingredients was 11.5% and the simultaneous effect with DSE species explained 13.8% of variation, which might be attributed to the effect of DSE species. Therefore, the interaction between residues and DSE can positively promote the growth of liquorice, whereas DSE might be the primary factor affecting the active ingredients.

## Discussion

Despite the ubiquitous occurrence of DSE, their behavior in plant roots and hence their ecological roles on plant performance are poorly investigated (Mandyam and Jumpponen, 2005; Berthelot et al., 2016). This study reported the first record of DSE colonization in liquorice roots. In all the inoculated liquorice plants, we observed typical DSE hyphae and microsclerotia in roots, indicating that these DSE are effective root colonizers. The SEM analysis and variance partitioning showed that DSE significantly and directly influenced plant biomass, root surface area, and plant active ingredient and mineral nutrient content; DSE is the primary factor affecting the growth and active ingredients of liquorice. This was consistent with the findings of previous studies that DSE affect the growth and medicinal quality of medicinal plants (Wu et al., 2010; Zhang et al., 2012; Zhu et al., 2015). Meanwhile, to our knowledge, this study is the first to report the positive influence of *P. putaminum* as a plant pathogen on plant growth (Jiang et al., 2017). Moreover, the higher root mass probably indicates the higher presence of lateral and secondary roots and root hairs, which are morphological adapted root characteristics for more efficient uptake of nutrients (Jumpponen and Trappe, 1998; Narisawa et al., 2007). In the present study, in plants subjected to DSE inoculation, the plant N and P content increased, but the soil organic matter and available P content in rhizosphere soil decreased, probably due to their remarkable enhanced uptake by the host plant, which has also been observed in previous studies (Alberton et al., 2010; Orujeia et al., 2013; Li et al., 2018).

In this study, the addition of liquorice residue significantly improved the soil mineral nutrient, plant growth, and nutrient status. It is well known that residues after proper microbial fermentation can act as good organic amendments and can be used as a soil conditioner or organic fertilizer for cultivating plants (Zerpa et al., 2010; Rashid et al., 2016; He et al., 2017; Vimal et al., 2017). Krawczyk et al. (2017) found that waste substrates create a favorable root environment for the examined species and can significantly influence the plant growth and element concentration. Scalise et al. (2017) also found that the addition of residue affects the soil C pools and root development. Meanwhile, in our study, the variance partitioning and SEM analysis results showed that the interaction between residue and DSE can positively influence the soil mineral nutrient content, plant biomass, root surface area, glycyrrhizin content, and plant N and P content. Similar to the present study results, Duan et al. (2011) reported that the additional residue promoted the growth and total P uptake in plants inoculated with the AMF in disturbed soil, compared with that in plants treated with no residue. Moreover, in this study, compared with that in plants treated with sterilized residue alone, *P. putaminum* combined with sterilized residue significantly increased the soil organic matter and available N content, whereas, *A. vagum* combined with sterilized residue significantly increased the soil available N and available P content. These findings showed that DSE fungi perhaps act as residue decomposers. It has been reported that several complex macromolecular compounds can be used by DSE fungi (Caldwell et al., 2000). As a bridge linking the plant and soil environment (Peterson et al., 2008), DSE fungi increases the interaction between plants and soil and expands the pool of N and P that can be used by host plants because they secrete lots of enzymes to mineralize organic N and insoluble P in soil into available forms; thus, promoting the growth and tolerance of plants to stressful conditions (Newsham, 2011; Monica et al., 2015). Although the degradation of specific organic nutrient sources can differ among different DSE species (Della Monica et al., 2015), studies have suggested that DSE fungi can mineralize organic compounds that contain N and P, improving their availability to plants (Baum et al., 2018). A recent study using *Asparagus* demonstrated that *Phialocephala fortinii* isolates can convert soil organic nutrients to available forms to support the plant growth. The ability of *P. fortinii* to degrade C and N organic compounds suggests that this fungus might play roles in providing nutrients to host plants via organic matter decomposition (Surono and Narisawa, 2017). However, the mechanisms involved in increasing the growth of plants inoculated with DSEs require further investigation.

The difference between unsterilized and sterilized residues is that unsterilized residue contains *T. viride* population. In this study, the addition of unsterilized residue increased the soil organic matter content and decreased the soil available P and available N content, resulting in an increase in plant N and P content, compared with those of the plants treated with sterilized residue. *T. viride* has been widely considered as an effective biofertilizer, soil amendment, and biocontrol agent (Ahuja and Bhatt, 2018; Khan and Parveen, 2018; Bayoumi et al., 2019). Wang et al. (2018) reported that *T. viride* biofertilizer can improve the transfer of ammonium from soil to sweet sorghum and increase the dry weight of plants. The capacity of *T. harzianum* to solubilize inorganic nutrients and to increase nutrient uptake of plants has been demonstrated in several studies. It has also been hypothesized that P can be solubilized and stored in *Trichoderma*, to be released in a readily available form close to the roots after the lysis of aged mycelium (Altomare et al., 1999). A possible reason to explain this funding might be the relationship between AMF and *Trichoderma* sp. modification of root exudates caused by the AMF (McAllister et al., 1995). Furthermore, substances released by the extrametrical mycelia of AMF might also affect microbial populations (Filion et al., 1999; Martínez-Medina et al., 2009). Alpa et al. (2015) reported that AMF in combination with *T. viride* enhances nutrient alimentation especially P and plant growth hormones, leading to an improved rhizospheric condition in soil, influencing the physiological and biochemical properties of *Helianthus annuus*. These findings suggest a complex interaction between DSE and *T*. *viride.* According to the variance partitioning analysis, 57.5% of unexplained variables in the growth index and 26.7% of unexplained variables in active ingredient content still persisted, suggesting that there are still several unexplored factors that might also significantly affect the growth and active ingredient accumulation of liquorice, such as DSE inoculation size, plant culture time.

## Conclusion

In this study, we first reported DSE associations with liquorice and had a positive effect on plant growth, and glycyrrhizic acid and glycyrrhizin accumulation of liquorice depending on DSE species. DSE fungi inoculation can facilitate the nutrients absorption by improving the effectiveness of soil nutrition and structure of the root system, in order to promote the growth of plants and increase the level of active ingredient. Furthermore, the combination of DSE and residue enhanced plant growth more effectively than that with either agent applied alone. Future research should consider the resource and function of DSE associations in different plant species to improve the understanding of the role of DSE fungi in medicinal plant cultivation and waste materials utilization.

## Data Availability

All datasets generated and analyzed for this study are included in the manuscript and/or the Supplementary Files.

## Author Contributions

CH and WW conceived and designed the experiments. CH performed the experiments. CH and JH analyzed the data. CH and WW wrote the manuscript.

## Conflict of Interest Statement

The authors declare that the research was conducted in the absence of any commercial or financial relationships that could be construed as a potential conflict of interest.
