# Supplementary material for: Characterization of Dark Septate Endophytic Fungi and Improve the Performance of Liquorice Under Organic Residue Treatment
Source: Front Microbiol. 2019 Jun 18;10:1364. doi: 10.3389/fmicb.2019.01364 (PMC6592127; doi:10.3389/fmicb.2019.01364)
Supplement: Supplementary file 1 [file Table_1.DOC]

**Table S1** HPLC mobile phase and gradient elution.

| Time (min) | A (acetonitrile) | B (deionized water : phosphoric acid) |
| --- | --- | --- |
| 0.0 | 14% | 86% |
| 10.0 | 23% | 77% |
| 24.0 | 30% | 70% |
| 30.0 | 34% | 66% |
| 35.0 | 36% | 64% |
| 42.0 | 42% | 58% |
| 48.0 | 51% | 49% |
| 60.0 | 14% | 86% |

**Table S2** Mantel tests showing correlationships (R values) between DSE, residue, total biomass, root surface area, glycyrrhizic acid, glycyrrhizin, plant total N, plant total P and soil nutrient elements content.

| Variable | DSE | Residues | DR | TB | TSA | GAC | GC | PTN | PTP | SOM | SAP | SAN |
| --- | --- | --- | --- | --- | --- | --- | --- | --- | --- | --- | --- | --- |
| DSE | 1 |  |  |  |  |  |  |  |  |  |  |  |
| Residues | -0.034 | 1 |  |  |  |  |  |  |  |  |  |  |
| DR | 0.263*** | 0.118** | 1 |  |  |  |  |  |  |  |  |  |
| TB | 0.530*** | 0.173** | 0.889*** | 1 |  |  |  |  |  |  |  |  |
| TSA | 0.170*** | 0.184** | 0.257*** | 0.496*** | 1 |  |  |  |  |  |  |  |
| GAC | 0.0887* | 0.197** | 0.157** | 0.473*** | 0.385** | 1 |  |  |  |  |  |  |
| GC | 0.826* | 0.2511*** | 0.147** | 0.032 | -0.114 | -0.055 | 1 |  |  |  |  |  |
| PTN | 0.282*** | 0.278*** | 0.161** | 0.031 | -0.015 | 0.013 | -0.109 | 1 |  |  |  |  |
| PTP | 0.267*** | 0.052 | 0.21** | 0.153* | 0.094 | -0.023 | -0.078 | 0.544*** | 1 |  |  |  |
| SOM | -0.230* | 0.595*** | 0.136** | 0.220*** | 0.268** | 0.112 | -0.163 | 0.054 | -0.052 | 1 |  |  |
| SAP | 0.049 | 0.307*** | 0.531*** | 0.064 | 0.037 | 0.083** | -0.109 | -0.018 | -0.245** | -0.365*** | 1 |  |
| SAN | -0.211* | 0.118* | 0.356*** | 0.353*** | 0.069 | 0.373*** | -0.129 | -0.079 | -0.104 | 0.159* | 0.247*** | 1 |

DR: DSE+Residues; TB: Total biomass; TSA: Root surface area; GAC: Glycyrrhizic acid content; GC: Glycyrrhizin content; PTN: Plant total N; PTP: Plant total P; SOM: Soil organic matter; SAP: Soil available P; SAN: Soil available N. **P* < 0.05; ***P* < 0.01;****P*<0.001.
